# New Scheme of MEMS-Based LiDAR by Synchronized Dual-Laser Beams for Detection Range Enhancement

**DOI:** 10.3390/s24061897

**Published:** 2024-03-15

**Authors:** Chien-Wei Huang, Chun-Nien Liu, Sheng-Chuan Mao, Wan-Shao Tsai, Zingway Pei, Charles W. Tu, Wood-Hi Cheng

**Affiliations:** 1 Department of Electrical Engineering, National Chun Hsing University, Taichung 402, Taiwan; cwhuang6374@dragon.nchu.edu.tw (C.-W.H.); r520530570r@gmail.com (S.-C.M.); wstsai@nchu.edu.tw (W.-S.T.); ctu@ucsd.edu (C.W.T.); 2 Graduate Institute of Optoelectronic Engineering, National Chun Hsing University, Taichung 402, Taiwan; zingway@dragon.nchu.edu.tw

**Keywords:** MEMS LiDAR, auxiliary laser, long detection range

## Abstract

A new scheme presents MEMS-based LiDAR with synchronized dual-laser beams for detection range enhancement and precise point-cloud data without using higher laser power. The novel MEMS-based LiDAR module uses the principal laser light to build point-cloud data. In addition, an auxiliary laser light amplifies the single-noise ratio to enhance the detection range. This LiDAR module exhibits the field of view (FOV), angular resolution, and maximum detection distance of 45° (H) × 25° (V), 0.11° (H) × 0.11° (V), and 124 m, respectively. The maximum detection distance is enhanced by 16% from 107 m to 124 m with a laser power of 1 W and an additional auxiliary laser power of 0.355 W. Furthermore, the simulation results show that the maximum detection distance can be up to 300 m with laser power of 8 W and only 6 W if the auxiliary laser light of 2.84 W is used, which is 35.5% of the laser power. This result indicates that the synchronized dual-laser beams can achieve long detection distance and reduce laser power 30%, hence saving on the overall laser system costs. Therefore, the proposed LiDAR module can be applied for a long detection range in autonomous vehicles without requiring higher laser power if it utilizes an auxiliary laser light.

## 1. Introduction

In recent years, light detection and ranging (LiDAR) [1] has become the crucial sensing unit in autonomous driving and environmental detection because it can build 3D information by detecting the object distance, air quality, and topography [2]. A LiDAR mainly uses a laser beam passing through a micro-lens to distribute the average matrix spot onto a target. The reflected light passes through a focusing micro-lens to an avalanche photodiode (APD), and a microprocessor calculates the object distance and produces a 3D point cloud. However, the intensity of the reflected signal strongly depends on the laser power and the responsiveness of the APD to light. Enhancing LiDAR detection distance requires a high-power laser, a high-sensitivity detector, and a complex receiving system. However, these approaches can make LiDAR systems bulky and expensive. Therefore, a LiDAR system needs to have a simple architecture to achieve long-distance ranging, high resolution, and precise point-cloud data [3,4,5,6].

We propose a novel scheme of MEMS-based LiDAR using synchronized dual-laser beams for the first time. The synchronized dual-laser beams are based on optical stochastic resonance theory to enhance the sensitivity of the LiDAR [7]. The visual signal-to-noise ratio condition shows that noise plays a constructive role in signal processing [7,8,9,10]. The detector can detect weak time-varying signals in a noisy environment when the intensity of the noise signal reaches a limit at an appropriate level. To enhance the LiDAR detection distance and analysis that our team studies, we refer to the synchronous detection circuit on the photodetector established by Liu et al. [11], and the stochastic resonance theory explored by Dodda et al. [12] serves as the research foundation for our research team. Dodda et al. showed that introducing a small but ideal quantity of external signals can enable the detection of sub-threshold signals. Noise can come from weak periodic signal sources or be added externally through on-chip noise generators. Liu et al. could easily detect faint light signals using a synchronous detection circuit comprising a switching phase amplifier, a low-pass filter, and a trans-impedance amplifier. Also, Table 1 contrasts additional techniques for improving image resolution and detection range. We analyze using items 1 and 2′s architecture as a basis and address its flaws. Items 3 to 5 can swiftly swap out the original parts, but the size and pricing will impact the cost and application of the LiDAR.

In this study, we demonstrate the field of view (FOV), angular resolution, and maximum detection distance to be 45° (H) × 25° (V), 0.11° (H) × 0.11° (V), and 124 m, respectively. The maximum detection range of the LiDAR is increased by about 16% from 107 m without the auxiliary laser to 124 m with one. The FOV boundaries are clear, and the enhanced recognition intensity has low signal noise. In addition, the simulation result shows that the maximum detection range can be more than 300 m with an auxiliary laser. Therefore, the proposed MEMS-based LiDAR by synchronized dual-laser beams with excellent optical performance may be promising as a candidate for use in autonomous driving, surveillance, and fast 3D modeling applications without using higher laser power.

## 2. Structure of MEMS-Based LiDAR

### 2.1. Scheme of MEMS-Based LiDAR

Using the optical stochastic resonance theory can increase the detection distance of a LiDAR with two laser sources. The two light sources are incident on the APD sensor at a certain angle, and the secondary light source will couple and transfer the energy to the main light source. An amplifier will amplify the final light signal. Use two independent light sources to replace the split light sources. One can obtain a longer detection distance when one can minimally adjust the output power of the auxiliary light source in the original architecture.

**Figure 1:**
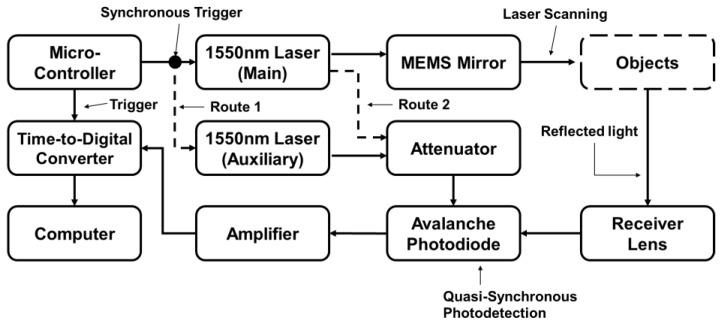
Block diagram of the MEMS-based LiDAR system with synchronized laser beams.

Figure 1 shows a diagram of a MEMS-based LiDAR with synchronized dual-laser beams. A microcontroller (MCU) manipulates the electrical signal to control the entire system. The synchronized signals trigger two separate lasers, and the power density of the auxiliary laser is lower than that of the main laser. The APD detects the reflected light from the main and auxiliary light and converts it into an electrical signal under test. A trans-impedance amplifier (TIA) circuit converts the current signal into a voltage signal and feeds it to a time-to-digital converter (TDC) circuit to calculate the flight time. The trigger signal from the MCU and the detected light signal are the two inputs for the TDC.

**Figure 2:**
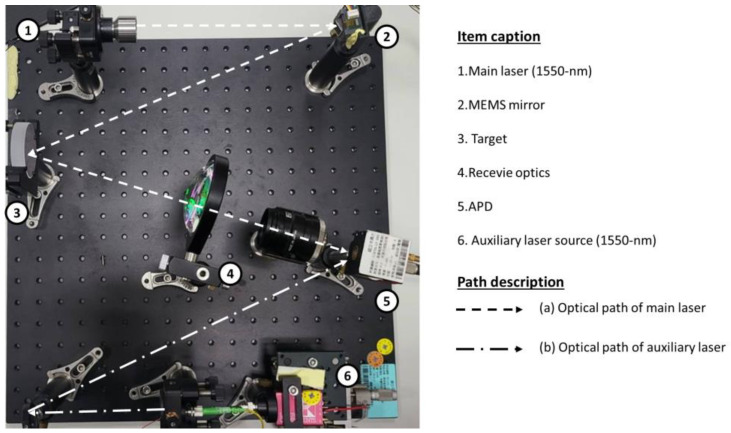
A simple verification architecture of an optical path for the synchronized dual-laser beams. The path of the dashed line is the detection optical path from the main laser source, MEMS mirror, and receiver optics to APD. The path of the dash-dotted line is the enhanced signal optical path from the auxiliary laser source directly reaching the APD.

To validate the theoretical concept, we established a straightforward verification architecture on an optical board, as illustrated in Figure 2. This architecture consists of a primary optical detection path equipped with a laser source and a MEMS mirror, a receiving optical path designed to capture the optical signal using a receiving lens and an APD, and an auxiliary light source to enhance the sensitivity and intensity of the signal. The optical path fixes the transmission and reception angles by adjusting the intensity of the laser source during the verification process. In the verification mode, connect an oscilloscope to verify that the signal exceeds the threshold and utilize the point-cloud image module to confirm resolution improvements, as detailed in Section 2.2 and Section 3.1.

### 2.2. Theory of Dual-Laser Sources to Enhance the Detection Distance

**Figure 3:**
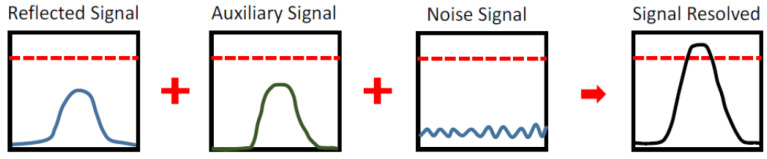
The schematic of auxiliary light amplifies the reflected signal optically. The solid line represents the signal value, and the dotted line represents the threshold value.

A schematic of the proposed dual-beam optical amplification scheme is shown in Figure 3. This technique affects the LiDAR system when the incident light collected by the focus lens to the PD is too weak to be resolved by the receiver system. The PD receives the light signal reflected by an object in front of the LiDAR system. Suppose the object has low reflectance, such as a distant target, glass, rubber tire, or a tilted-angle flattened surface. In that case, the collected signal level may be below the sensitivity threshold of the receiver system. An auxiliary light signal illuminates the PD directly to amplify this signal. This auxiliary signal is synchronized to the primary laser beam and arrives at the PD simultaneously with the reflected primary signal. The optical intensity exceeds the receiver’s sensitivity threshold with the auxiliary laser beam amplification. Moreover, if the laser beam from the LiDAR system does not reach an object in a specific direction, there is no reflected light. In this case, the PD receives only environmental noise. There should not be a reading by the PD. In addition to the noise signal, with an adequate intensity for the auxiliary laser beam, the received light intensity of the PD will not exceed the sensitivity threshold of the receiver.

The auxiliary light signal has a gain impact on the weak signal in the reflected signal after the reflected signal and auxiliary light signal enter the quasi-synchronous APD, allowing the weak signal’s intensity in the reflected signal to exceed the APD threshold. This study uses stochastic resonance to boost the weak signal’s intensity, and the main laser signal and the auxiliary laser signal’s time of entry into the APD perfectly sync. 

**Figure 4:**
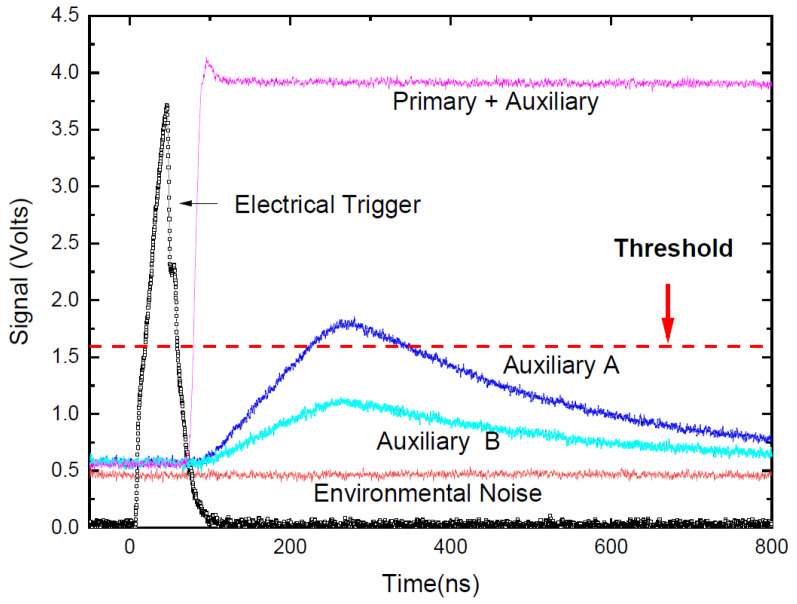
The time sequence of electrical trigger signal, primary and auxiliary lasers, and environmental noise obtained by an oscilloscope. To exceed the threshold, the power coupling of the main light and the auxiliary light must rely on an attenuator to adjust the power of the auxiliary light (A and B).

This study used a MEMS-based LiDAR with a 1550 nm laser (LS5-5-4-S10-00) that operates at a 1 MHz repetition rate. The thermopile sensor (919P-003-10, Newport) shows 54.3 mW when it measures the average power of the main laser. To confirm the amplification of the synchronized dual-laser beams in the verification architecture, we utilized a high-data-rate oscilloscope to showcase the time sequence of the electrical trigger and received signal, as illustrated in Figure 4. Figure 4 displays the time sequence of the electrical trigger signal and primary and auxiliary lasers from a high-data-rate oscilloscope. There are two electrical triggers synchronized from the MCU: one is used to trigger the 1550 nm laser, and the other is connected to the oscilloscope. In addition, the primary and auxiliary lights are closely coupled to the APD to detect the optical intensity. An attenuator tunes the power of the auxiliary light to prevent confusion with the actual signal. Two levels of attenuators are used, denoted as A and B. The oscilloscope also records the noise of APD exposure to the environment. The electrical trigger is predominant, with a trigger time around the origin. Together with the auxiliary laser, the primary laser is of exceptionally high intensity. The oscilloscope signal from the APD exhibits a rapid increase, with around 70 ns delayed to the electrical trigger signal. The response time of the laser driver circuit, optical system, and APD causes this delay. Moreover, a series of calibrations are required to obtain the actual ToF by removing the delay time. The APD measures the intensity of the auxiliary laser after attenuators A and B and feeds the result into the TDC circuit and oscilloscope separately. The power of auxiliary A is higher than the threshold of the TDC circuit that reads out the distance. The TDC circuit connects the APD signal from auxiliary B without resolving a distance readout that verifies the feasibility. Therefore, auxiliary B is feasible for the subsequent measurement. Furthermore, the environmental noise shows a deficient signal level that would not cause any measured distance in the LiDAR system.

**Figure 5:**
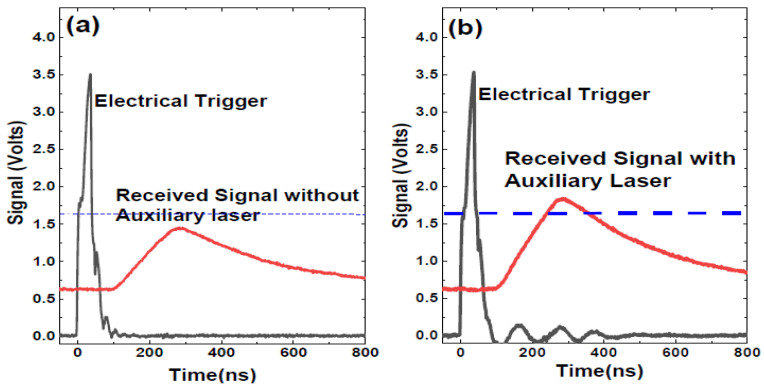
The oscilloscope signal is directly from an APD on detecting reflected light in two types of LiDAR architecture: (**a**) without an auxiliary laser light and (**b**) with auxiliary laser illumination. The red solid line displays the value of the light signal, and the dotted line displays the value of the threshold.

We stretched the first test object from 15 to 30 cm during the target test. It is visible through the picture of the point cloud. The scanning laser reflects the APD, causing the actual light intensity to fall below the APD trigger point and preventing its generation. The auxiliary light laser is activated, forcing the signal initially near the APD trigger point to rise above the trigger and produce a ToF value. This method dramatically improves the graphics resolution of the object. Figure 5a shows the received signal without an auxiliary light, in which the intensity is lower than the threshold, and the distance is unresolved. The system determines the threshold, considering the APD sensitivity, TIA circuit, and TDC limit. Figure 5b shows the intensity exceeding the threshold when the auxiliary light amplifies the reflected signal. 

## 3. Measurements and Results

### 3.1. Measurement of the Verification Architecture for the Synchronized Dual-Laser Beams

**Figure 6:**
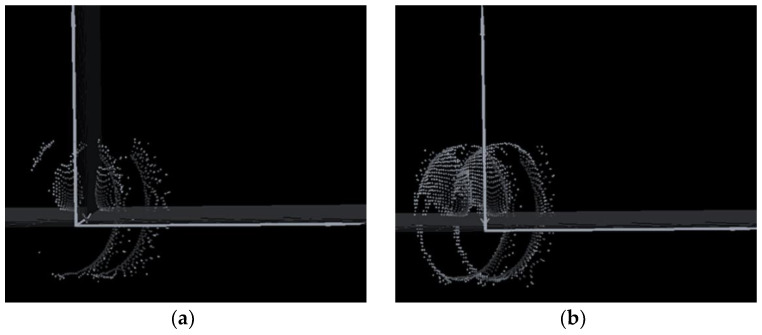
The point-cloud image from APD catches the received optical signal from the target (**a**) without an auxiliary laser light and (**b**) with auxiliary laser illumination. As seen by the image, under the identical detection effect, there are far more point clouds with the auxiliary light system turned on than without. Additionally, the edge features of the target object as a whole are made evident.

This work detects the target on the optical board when the verification architecture activates or deactivates the auxiliary laser light. As illustrated in Figure 2, the auxiliary laser directly irradiates the APD while simultaneously scanning with the main laser source. The APD remains unaffected by the auxiliary laser because it synchronously operates with the signals for both the APD and the main scanning laser. Consequently, the overall scanning resolution enhances the threshold signal in the APD when the auxiliary laser injects the appropriate luminous flux. The action function and resolution of the point-cloud image are presented in Figure 4 and Figure 6, respectively.

In Figure 6a, the point-cloud image of the target is unclear because the intensity of reflected light is below the trigger point of the APD, and the auxiliary laser light system is inactive. The graphic resolution in Figure 6b significantly improves the recognition of objects and contours when activating the auxiliary laser light system. This result explains how the auxiliary laser system enhances the resolution in this verification architecture by forcefully elevating the signal beyond the original intensity. Subsequently, we construct a MEMS-based LiDAR with an auxiliary light system for practical measurements.

### 3.2. Measurement of MEMS-Based LiDAR

**Figure 7:**
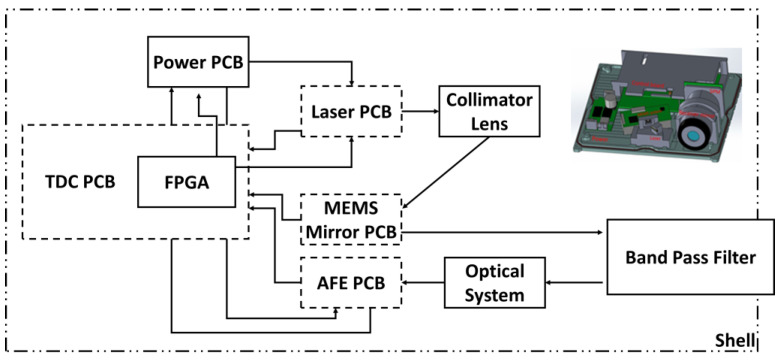
The architecture of the hardware configuration for the MEMS-based LiDAR. Under the casing, the entire LiDAR structure covers the laser light source group, optical lens group, computer circuit board, electronic control circuit board, and power control circuit board. We set up every component grid using space optimization and visual route design.

Figure 7 shows a MEMS-based LiDAR module composition. The launch system components include a 1550 nm laser diode with a driver board, a MEMS with a driver board (UM-6002F, Ultimems Inc., New Taipei City, Taiwan), and a collimator lens. The receiving system components comprise an APD (A-CUBE-l200-01) with a driver board and a receiver lens. The laser control, MEMS control, signal transfer, and point-cloud processing have used the field programmable gate array (FPGA, Xilinx, San Jose, CA, USA). The redesigned circuit board has added a high-speed operational amplifier to stabilize the signal and increase the width of the input signal in the signal port. The agency design target ensures optical stability, reliability, and functionality, which have two main items in the mechanism design of internal components and casing design. The mechanism design of internal components is the distance and placement, especially the spacing, height, and angle of incidence between the laser and MEMS. In addition, when we set all of them, we consider the intersection of the electric wire, wiring, and cell. The last step is designing a case to avoid collisions with the internal components and the inside power. The material of the shell is aluminum to release the heat. Finally, the window adds a bandpass filter to pass specific wavelengths, avoid interference with sensing parts, and reduce reflectivity at particular wavelengths.

### 3.3. Measurement of FOV, Angular Resolution, and Maximum Detected Distance

**Figure 8:**
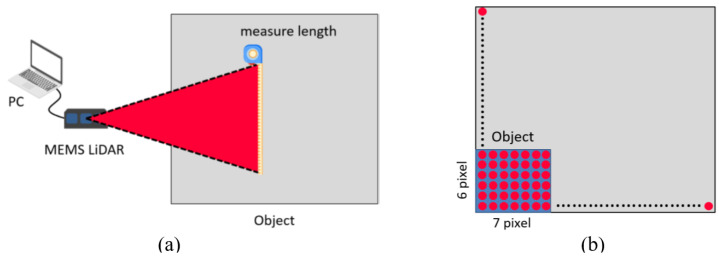
The method and analysis describe the FOV and angular resolution in MEMS-based LiDAR. Conduct the angular resolution test in the situation shown in (**a**). Configure the environment in compliance with the requirements of the standard test. Use trigonometric functions and point-cloud data to compute the angular resolution; (**b**) although the transmitted object’s actual size is equal to the proportionate relationship of the site size, users can utilize this information to estimate the approximate number of point clouds.

We investigated the FOV, angular resolution, and maximum detected distance for the MEMS-based LiDAR. We calculated the FOV and angular resolution values in different detection ranges. Figure 8 shows the architecture and method for the MEMS-based LiDAR. 

The MEMS-based LiDAR operated with its original output parameters, including a power output ratio of 70%, a frequency of 1 MHz, and a frame rate of 10 fps. We calculated the FOV with the distance between the LiDAR and the object, length, and width of the point-cloud image when the detection range was 280, 380, and 480 cm. Table 2 lists the measurement data and specifications of MEMS-based LIDAR. Table 2 indicates that the FOV of MEMS-based LiDAR is 45° ± 2° (H) × 25° ± 2° (V), and the tolerance value conforms to the specification for the design value of the MEMS unit. The angular resolution calculated from the image data of FOV depends on the scanning angle of each point-cloud value. Therefore, the angular resolution is calculated as 0.11° (H) × 0.11° (V) when the tolerance value conforms to the specification for the design value.

### 3.4. Results and Discussions

In the university campus environment, we perform the detection range measurement of the MEMS-based LiDAR with the original output data parameters. The test environment consists of a straight road at night with buildings and vehicles present, as shown in Figure 8a. We placed the LiDAR in the middle of the road. The measurement involved moving the target to the maximum detected range and performing lateral movement to ensure accurate detection of the target, which in this case was a human. The target object in this test, a pedestrian, had an average reflectivity of 18%.

According to the theory and format of APD [16], the point-cloud image does not show potential signal noise under the intensity of the signal below the threshold of APD when the output power ratio of the auxiliary light is below 20%. The point cloud and boundaries were not fully clear when the LiDAR generated them without the low intensity of the auxiliary laser. Although there are a more significant number of point clouds visible than in the original image when the image output power ratio of the auxiliary light is 20%, the boundaries of the FOV angle are not fully displayed. There has been evidence of epitaxy boundaries at the FOV angle and lower signal noise when the power output ratio of the auxiliary light is over 30%. 

**Figure 9:**
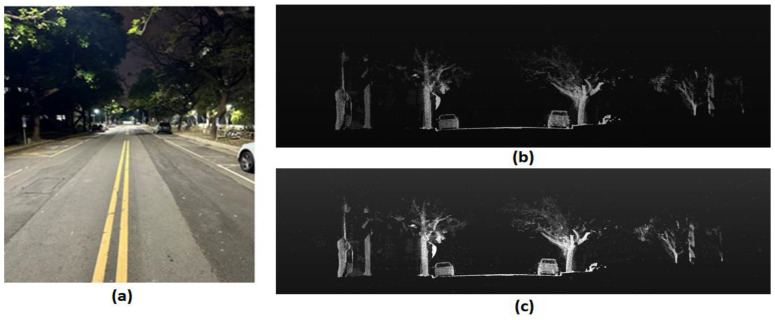
The method and analysis description for the FOV and angular resolution in MEMS-based LiDAR: (**a**) is the optical image of the test environment; (**b**) is the point-cloud image without the auxiliary laser light; and (**c**) is the point-cloud image with the auxiliary laser light. The picture (**c**) shows more point clouds, project contours, and weak signals from objects farther behind.

Figure 9b,c shows the front view of the point-cloud image of a scene on our university campus without and with auxiliary light, respectively. The image in Figure 9c is more evident than in Figure 9b. Table 3 lists the measured maximum detection range of the LiDAR module with different powers of the auxiliary light from 0 to 355 mW. The total detection range of LiDAR is enhanced by about 16% from 107 m without an auxiliary laser to 124 m with one for the laser output power of 1 W and the auxiliary power of 355 mW, with the power ratio being 35.5%. The results demonstrate that enough energy from the auxiliary light can effectively improve the detection performance of LiDAR. However, the maximum detection range could be more than 300 m if the laser output power is 6 W with auxiliary light. We will discuss this setup in simulation Section 3.5.

### 3.5. Simulation Results

We investigate simulations with higher laser power ranging from 1 to 10 W to increase the detection distance. For simulation, the maximum detection distance, a laser injection power, an object reflective rate, and the received power of the laser signal are used [17]. A simulation equation is given by

(1) $$P_{r}=\frac{\alpha P}{R^{2}}$$

where Pr is the received power for the laser signal, P is the laser inject power, R is the detection distance, and α is the object reflective rate.

**Figure 10:**
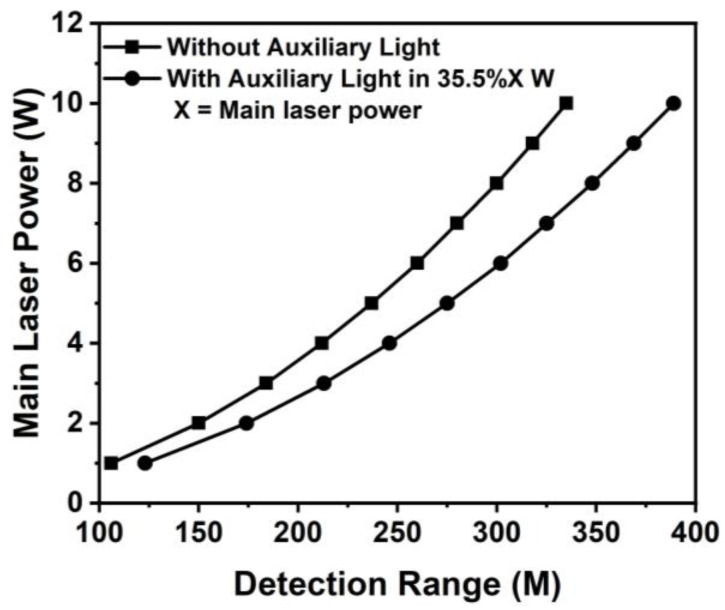
The maximum detection range is a function of the incident main laser power. The LiDAR presents the detection range by the line of dots when activating the auxiliary light system. The auxiliary light power is 35.5% of the leading laser power, and the squares line represents when the auxiliary light system is off. The detecting distance will be 16% farther when the auxiliary light system is active than when it is inactive.

Figure 10 shows the simulation result of the maximum detection distance as a function of laser power with and without auxiliary laser light. The auxiliary laser power is 35.5% of the main laser power. The maximum detection distance is enhanced by 16% from 107 m to 124 m with a laser output of 1 W, and an additional auxiliary laser power of 0.355 W. Figure 6 indicates that the maximum detection ranges are estimated to be 212 and 300 m for laser powers of 4 and 8 W, respectively. The total detection ranges are up to 246 and 348 m as the LiDAR adds auxiliary lights of 1.42 W and 2.48 W, respectively. The simulation results show that the maximum detection could be up to 300 m with a laser power of 8 W but still only 6 W with an auxiliary laser light of 2.84 W. Therefore, the synchronized dual-laser beams can reduce laser power 30% and, hence, save costs for the overall laser system. With an auxiliary laser power of 10 W, the system expects to achieve a maximum detection distance of 389 m.

After adding the benefits of this auxiliary light architecture above, we can contrast its cost, power consumption, and structural volume with LiDARs that do have/do not have auxiliary lightweight architecture and have a 300-m detection range. Refer to Table 4 for comparison.

We provide different explanations for the pricing, structural volume, and power loss as follows:

1. Price: A high-power laser chip with a wavelength of 1550 nm and a power of more than 4 W is not a product that one can commercially purchase. This product costs more than the standard model.
2. Power Consumption: With or without an additional laser, the LiDAR’s overall output power was smaller. In general, the main laser’s consumption rate was smaller. However, when directly pumping the auxiliary laser system into the APD when the range is very close, there is very little loss of advantage.
3. Dimension: The high-power laser module was more extensive compared to the low-power one. Therefore, this will impact the module structure and other electrical component parameters. This specification indicates that the volume will be larger than low-power LiDAR. Additionally, as the auxiliary laser system is an additional laser light source attached to the existing structure, with an input direction of light from the outside to the inside, it requires extra room. In general, there will be little difference between the two volumes.

## 4. Conclusions

We report a new scheme of MEMS-based LiDAR by synchronized dual-laser beams for detection range enhancement without using a high-power laser source. The LiDAR module may exceed the APD receiving limit and cause a short circuit in the LiDAR when the output power of the auxiliary light is over 400 mW. One may expect the detection distance to increase to over 300 m with component improvement. The maximum detection range may increase to 389 m by employing dual-laser beams when the MESM-based LiDAR carries an auxiliary light system.

In summary, we have demonstrated a new scheme of MEMS-based LiDAR by synchronized dual-laser beams to enhance detection range. The proposed MEMS-based LiDAR exhibits a FOV, angular resolution, and maximum detection distance of 45° (H) × 25° (V), 0.11° (H) × 0.11° (V), and 124 m, respectively. The maximum detection range of the LiDAR is enhanced by about 16% from 107 m without an auxiliary laser to 124 m with one. The synchronized dual-laser beams optically amplify the reflective signals. We use the auxiliary light to boost the light signal beyond the resolving limit of the receiver system. The experiments indicate that amplifying the signal with the auxiliary light can achieve a uniform distance distribution on a low-reflectivity background and a clear image at the object’s edge. The results show that achieving enhanced maximum detection ranges, clear FOV boundaries, reduced power requirements, and intensified recognition in low signal noise is possible when reaching the optimal output ratio of the auxiliary light. Users can utilize the LiDAR module with a lower laser power for autonomous driving, surveillance, and fast 3D modeling applications, demonstrating these improved abilities.

## Figures and Tables

**Table 1:** Compare different ways to enhance point-cloud images or distances.

Item	Model	Advantage	Shortcoming
1 [11]	The dual-light source effect causes a stochastic resonance system.	A single light source splits into two and adjusts itself to enhance the intensity of the light signal.	A larger volume reduces the detection distance.
2 [12]	A circuit module boosts the signal that is received.	Able to boost weak signals to the sensor’s lowest possible reception level.	Synchronous control will result in a momentary black space.
3 [13]	Increase the power of vertical-cavity surface-emitting laser (VCSEL).	Using light source array mode to boost optical power.	The cost is too high; light source module temperature is too high.
4 [14]	Using high-sensitivity avalanche photodiode (APD).	Developing low excess noise APDs with new material (GeSi).	In development.
5 [15]	Improve the optical lens modules’ matching.	Less of an effect on the LiDAR’s general structure.	Extremely exacting in terms of lens design and specs.

**Table 2:** FOV measurement results between measurement data of MEMS-LiDAR and specification of MEMS.

	Measurement Data			Specification of MEMS
Measurement distance (cm)	280	380	480	380
Length for field (cm)	240	320	400	NA
Width for field (cm)	132	163	218	NA
Horizon for FOV (°)	46.2	45.6	45.2	45
Vertical for FOV (°)	26.5	24.2	24.4	25
Resolution for object	36 × 76	27 × 61	22 × 47	NA
Resolution	392 × 218	393 × 216	400 × 223	NA
Angular resolution (H °) × (V °)	0.1 × 0.1	0.1 × 0.1	0.1 × 0.1	NA

**Table 3:** The maximum detected range is 1 W for different output powers of the auxiliary light.

The Output Power of the Auxiliary (mW)	Maximum Detection Range (m)	Enhancement Rate (%)
0	107	X
0.1	113	6.7
53.6	115	8.5
204	116	9.5
355	124	16

**Table 4:** Comparison of LiDAR with/without auxiliary laser.

	Price	Power Consumption	Dimension
Without Auxiliary Laser	High	High	Smaller
With Auxiliary Laser	Low	Low	

## Data Availability

Data are contained within the article.
